# Measuring Mental Wellness of Adolescents: A Systematic Review of Instruments

**DOI:** 10.3389/fpsyg.2022.835601

**Published:** 2022-03-09

**Authors:** Zaida Orth, Faranha Moosajee, Brian Van Wyk

**Affiliations:** ^1^School of Public Health, Faculty of Community and Health Sciences, University of the Western Cape, Bellville, South Africa; ^2^Division for Postgraduate Studies, University of the Western Cape, Bellville, South Africa

**Keywords:** mental wellness, measurement, systematic review, adolescent, mental wellbeing

## Abstract

**Objective:**

Mental health is critical to the healthy development of adolescents. However, mental health encompasses more than the absence of mental illness; and should include indicators of mental wellness. A critical review of available mental wellness instruments for adolescents were conducted to identify operational definitions of mental wellness concepts for this population group.

**Method:**

A systematic review of literature published between 2000 and 2020 was done to identify mental wellness instruments for adolescent populations. The review followed the PRISMA operational steps.

**Results:**

We identified 2,543 articles from the search strategy and screened titles and abstracts for eligibility. After appraisal, 97 studies were included in the qualitative synthesis; of which, 79 mental wellness instruments were identified. Most studies did not provide a definition for mental wellness. We identified thirteen mental wellness concepts from 97 studies, namely: *life satisfaction, mental wellbeing [general], resilience, self-efficacy, self- esteem, connectedness, coping, self-control, mindfulness/spiritual, hope, sense of coherence, happiness*, and *life purpose.*

**Conclusion:**

The review reflected previous research identifying a lack of consensus around the definitions of mental health, mental wellness, and mental wellbeing. This has implications for developing instruments for adolescents that adequately measure these constructs. Most of the instruments identified in the review were predominantly English and from developed countries. This indicates a need for instrument that are explicitly conceptualised and operationalised for adolescents in all their varied contexts.

## Background

Adolescents are prioritised in the global public health agenda because they play a central role in achieving the 2030 Sustainable Development Goals (SDGs) (World Health Organization, 2017; Guthold et al., 2019; Patalay and Gage, 2019). In 2019 UNICEF estimated that there were 1.2 billion adolescents (aged 10–19 years), which represents approximately 16% of the global population (UNICEF, 2020a). It is further estimated that approximately 50% of all mental disorders have their onset during adolescence (Patalay and Gage, 2019; Peters et al., 2019; UNICEF, 2020b). According to the World Health Organization (WHO) (World Health Organization, 2020), one in seven adolescents experienced a mental health condition in 2019. Poor mental health hinders healthy adolescent development and is associated with poorer health, social and economic outcomes across their lifetime (Patalay and Gage, 2019; Peters et al., 2019). Adolescent mental disorders represent a significant burden of disease on health systems, particularly in low- and middle-income countries (LMICs), where mental health services and resources are lacking (Peters et al., 2019; World Health Organization, 2020; Sorsdahl et al., 2021).

Early intervention and prevention programs are critical for the healthy development of adolescents. The SDGs, the Global Strategy for Women’s, Children’s and Adolescent’s Health, the Global Accelerated Action for the Health of Adolescents, and the Lancet Commission on Adolescent Health and Wellbeing highlight the gaps in research on adolescent mental health and argues for the need for valid high-quality data across different contexts to identify priorities and monitor progress in adolescent health (Joav Merrick, 2016; Patton et al., 2016; World Health Organization, 2017). A notable exception is the UNICEF’s *Measurement of Mental Health among Adolescents at the Population Level (MMAP)* programme, which aims to measure the prevalence of mental health disorders globally (UNICEF, 2020b). However, mental health is more than the absence of [mental] illness symptoms and should also include measures of wellness (Manderscheid et al., 2010; Ryff, 2013; Manwell et al., 2015; Eriksson et al., 2019). Research on *positive* mental health or mental wellness among adolescents is largely underdeveloped (Eriksson et al., 2019). To stimulate more research on mental wellbeing among adolescents, measures of mental wellness are needed (Glasner et al., 2020). To this end, we systematically reviewed mental wellness instruments used in research with adolescents and report on the mental wellness concepts that emerge from the identified instruments.

## Methods

The review was registered with PROSPERO (CRD42020186707) and the methods for this review have been explained in detail in the published protocol (Orth and van Wyk, 2020).

### Review Question

We identified the following research questions:

• What instruments are used to measure the mental wellness in adolescents?
• What dimensions of mental wellness were measured? What indicators were used?
• How is mental wellness conceptualised in the studies?

### Search Strategy

The search strategy was developed in consultation with the faculty librarian. The databased searched included Ebscohost (PsycArticles, Academic Search Premier), Cumulative Index of Nursing and Allied Health Literature (CINAHL), Educational Resource Information Center (ERIC), Medical Literature Analysis Retrieval System Online (MEDLINE) Google scholar and Sabinet. The following search terms were used: [(adolescent* OR teenage* OR young people OR youth) [AND] (psychological instrument OR measure* OR tool) [AND] (mental health OR mental wellbeing OR psychological wellbeing OR mental wellness) [AND] (psychometric*; reliability*; validity*)]. The search was concluded in December 2020.

### Inclusion and Exclusion Criteria

Our study selection was guided by the Population, Intervention, Comparison, Outcome and Time (PICOT) criteria (see Table 1). To be included in the review studies had to include adolescents aged 10–19 years and the instruments used had to have a focus on general mental wellness [relating to positive mental health]. Instruments aimed at measuring symptoms of mental illness or aiding clinical diagnoses were excluded. Studies which included samples of people outside of our age criteria were only included if the study had a clear focus on discussing adolescent mental wellness.

**TABLE 1:** PICOT.

Population of interest	Adolescents aged 10–19 years
Intervention of interest	Use a validated measuring instrument of mental wellness
Comparison interventions	Not applicable
Outcomes	Mental wellness
Time	2000–2020
Other considerations	Study designs: Quantitative method or mixed methods. Language: All

The time period of the search strategy was chosen due to the paucity of research in this area (Rose et al., 2017; Bentley et al., 2019; UNICEF, 2020b). Furthermore, the prioritisation of adolescent health and the focus on adolescent friendly services occurred after 2000 (World Health Organization, 2012).

### Review Procedure

We follow the preferred reporting item for systematic reviews and meta-analysis (PRISMA statement) results in the conduct of this review. The number of hits for each database was recorded and the citations were exported to Mendeley citation software. Following this, two reviewers (ZO & FI) independently reviewed all the titles and abstracts to assess which articles are appropriate for inclusion. The full-text articles of the included abstracts were downloaded and independently reviewed to determine which articles should be included for the final assessment. Conflicts were discussed, and where necessary, arbitrated by third reviewer (BVW).

### Quality Appraisal

Articles remaining after abstract screening were critically appraised using the SFS scoring system version D (Smith et al., 2015). This tool was developed to evaluate the methodological coherence and rigour of full text studies by providing scores on a generic set of categories. The SFS scoring system contains 29 questions covering the following sub-sections, namely: (1) *purpose of the measure*; (2) *methodological rigour*; and (3) *general considerations*. The overall quality of the study is scored as weak (0–25%), moderate (26–50%), strong (51–75%), or excellent (76–100%). Only articles that scored 51% and above in each of the abovementioned sub-sections were included in the final synthesis as this indicated that the articles were of a good quality.

### Data Extraction and Synthesis

A descriptive meta-synthesis approach was used to identify and describe the mental wellness instruments used among adolescent populations. The synthesis of information regarding each instrument was presented in tabular form to display relevant information (Gough et al., 2012). The article information was entered into an excel sheet and the sample characteristics, ages, sample size, distinctive population, languages of studies, and geographic location and purpose of the instrument were extracted.

### Ethics

This review, which is a sub-study of the first-author’s doctoral research project, received ethics clearance from the University of the Western Cape Biomedical Research Ethics committee (BM19/09/18).

## Results

**FIGURE 1:**
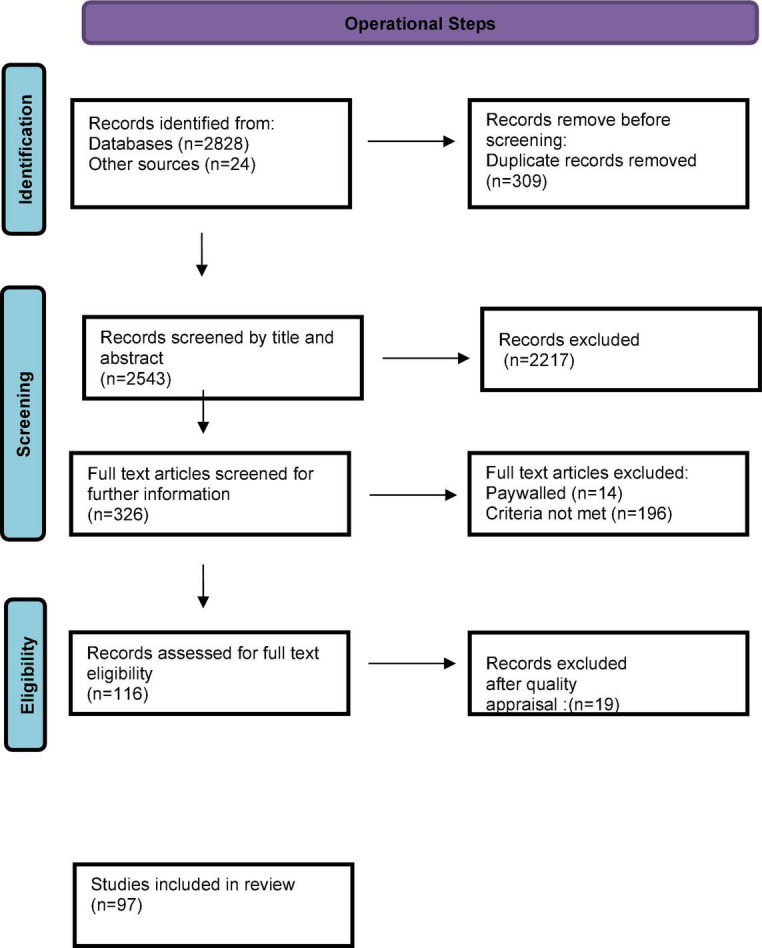
PRISMA flowchart for selection of studies.

The results of the screening and selection process are presented in the PRISMA diagram (Figure 1). We found 2,543 articles from the search strategy and screened titles and abstracts for eligibility; 326 full-text articles were screened for possible inclusion. We excluded 196 articles because the measures used in the study focussed on measures for mental illness or were conducted with general populations samples rather than adolescents specifically. Furthermore, 14 articles could not be accessed due to payment and were excluded. After conducting quality appraisal, a further 19 articles were excluded leaving a total of 97 studies to be included in the qualitative synthesis.

**FIGURE 2:**
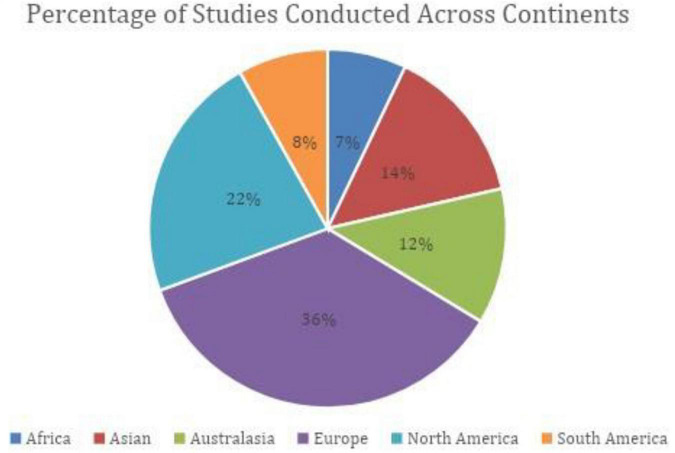
Pie chart depicting the percentages of studies conducted across continents.

As shown in Figure 2, most studies were conducted in Europe (36%) or North America (23%), and in English (*n* = 48); followed by Spanish (*n* = 18), Chinese (*n* = 8) and Portuguese language (*n* = 7). All instruments were originally developed in English; with some (*n* = 75) translated and adapted for use in a different cultural/language context.

Table 2 summarises the characteristics of 79 mental wellness instruments that were identified from the 97 studies. The instruments were categorised into 13 broad themes representing indicators of mental wellness namely, *life satisfaction* (*n* = 12), *mental wellbeing* [*general*] (*n* = 7), *resilience* (*n* = 7), *self-efficacy* (*n* = 7), *self-esteem* (*n* = 7), *connectedness* (*n* = 6), *coping* (*n* = 6), *self-control* (*n* = 6), *mindfulness/spiritual* (*n* = 6), *hope* (*n* = 5), *sense of coherence* (*n* = 4), *happiness* (*n* = 3), and *life purpose/goal* (*n* = 3).

**TABLE 2:** Summary of mental wellness concepts (*n* = 13) and mental wellness instruments (n-79).

Mental wellness concept	Definition of mental wellness	Title of instrument	Frequency of use; [Study references]
Mental wellbeing [general]	Experience of positive mental and physical health	Mental Health Continuum-Short Form	5; (Clarke et al., 2011; Guo et al., 2015; Carvalho et al., 2016; Piqueras et al., 2019; Reinhardt et al., 2020)
		Ryff Psychological Wellbeing Scale	5; (Gallardo Cuadra and Moyano-Diaz, 2012; Sirigatti et al., 2014; Kiang and Ip, 2018; Karen Meier and Beatriz Oros, 2019; ШУПЬГa, 2019)
		Warwick-Edinburgh Mental Wellbeing Scale (WEMWBS)	6; (Clarke et al., 2011; Hunter et al., 2015; McKay and Andretta, 2017; St Clair et al., 2017; Ringdal et al., 2018; Melendez-Torres et al., 2019)
		WHO-5 Wellbeing Index	5; (de Wit et al., 2007, 2012; Clarke et al., 2011; Carli et al., 2013; Stiglbauer et al., 2013)
		Warwick-Edinburgh Mental Wellbeing Scale short (SWEMWBS)	1; (McKay and Andretta, 2017; ШУПЬГa, 2019)
		EPOCH Measure (Engagement, Perseverance, Optimism, Connectedness, and Happiness)	1; (Kern et al., 2016)
		QEWB Eudaemonic Wellbeing Questionnaire	1; (Salavera and Usán, 2019)
Connectedness	Refers to the supportive and caring relationships of the child in relation to groups and other people	Hemingway Measure of Adolescent Connectedness–Short Version	1; (Snowshoe et al., 2017)
		Awareness of Connectedness Scale	1; (Peters and Peterson, 2019)
		Cultural Connectedness Scale-Short Version (CSS-S)	1; (Snowshoe et al., 2017)
		Milwaukee Youth Belongingness Scale (MYBS	1; (Slaten et al., 2019)
		Social Support Appraisals	1; (Slaten et al., 2019)
		Social Support Scale (Cluver)	1; (Gentz et al., 2018)
Coping	Ability to employ strategies to handle adverse or stressful events	A-COPE	1; (Forns et al., 2013)
		COPE	1; (Vaughn et al., 2009)
		KIDCOPE	1; (Powell et al., 2019)
		The Coping Response Inventory-Youth (CRI-Y)	1; (Erickson and Feldstein, 2007)
		The Schoolagers’ Coping Strategies Inventory (SCSI)	1; (Viñas et al., 2019)
		Utrechtse Coping List	1; (Mavroveli et al., 2007)
Self-control	Ability to control and regulate their emotions and thoughts	Difficulties in Emotion Regulation Scale (DERS)	2; (García-Alandete et al., 2019; Wang and Hawk, 2020)
		Trait Meta-Mood Scale (TMMS)	2; (Salguero et al., 2012; Ballespí et al., 2019)
		Weinberger Adjustment Inventory	2; (Vazsonyi et al., 2015; Slaten et al., 2019)
		Brief control scale	1; (Espada et al., 2019)
		Self-Control Scale (SCS)	1; (Black et al., 2012)
		Psychological Empowerment	1; (Ozer and Schotland, 2011)
Happiness	Emotional state of mind or mood that determines satisfaction with life, flourishing and overall, well being	Oxford happiness questionnaire	2; (Meleddu et al., 2012; Lung and Shu, 2020)
		Adolescent Happiness Scale	1; (Isik and Üzbe Atalay, 2019)
		The Subjective Happiness Scale (SHS)	1; (Gabrielsen et al., 2012)
Hope	Belief in the future and that hopes, and goals will be met	(FESA Scale) Children Future Expectation Scale	1; (Zhao et al., 2011)
		Children’s Hope Scale	2; (Ey et al., 2005; Ciarrochi et al., 2007)
		The Children’s dispositional hope scale	1; (Vaughn et al., 2009)
		The Hopefulness about Future (Hope) scale	2; (Zhao et al., 2011; Canfield et al., 2018)
		Urban Adolescent Hope Scale	1; (Canfield et al., 2018)
Life purpose/goal	Feeling that one’s life is significant, comprehensible, or purposeful	Adolescent Life Goal Profile Scale (ALGPS)	1; (Gabrielsen et al., 2012)
		Meaning in life questionnaire	1; (Shiah and Hwang, 2019)
		The Purpose in Life Test-10 Items	1; (García-Alandete et al., 2019)
Life Satisfaction	Overall quality of life rather than current feelings	Students’ Life Satisfaction Scale (SLSS)	5; (Furlong et al., 2014; Levin and Currie, 2014; Savahl et al., 2017; Burns and Rapee, 2019; Viñas et al., 2019)
		The Personal Wellbeing Index	4; (Casas et al., 2012, 2013; Sarriera et al., 2014; Tomyn and Weinberg, 2018)
		The Personal Wellbeing Index School Children (PWI-SC)	4; (Tomyn et al., 2013a,b, 2014; Viñas et al., 2019)
		The Satisfaction with Life Scale (SWLS)	6; (Gabrielsen et al., 2012; Shoshani and Steinmetz, 2014; Sanders et al., 2017; Snowshoe et al., 2017; Ringdal et al., 2018; Abujaradeh et al., 2020)
		Single Item on Overall Life Satisfaction	4; (Casas et al., 2012, 2013, 2014; Sarriera et al., 2014)
		Huebner’s Multidimensional Students’ Life Satisfaction Scale (MSLSS)	3; (Hatami et al., 2010; Sawatzky et al., 2010; Zeng et al., 2017)
		Cantril’s self-anchoring ladder	3; (Sawatzky et al., 2010; Levin and Currie, 2014; Melendez-Torres et al., 2019)
		Overall Life Satisfaction (OLS)	1; (Savahl et al., 2017)
		Affect Balance Scale (ABS)	1; (Wang and Hawk, 2020)
		Multidimensional Student Life Satisfaction Questionnaire Short Form (MDAS-SF)	1; (Duy and Yildiz, 2014)
		The Children’s Intrinsic Needs Satisfaction Scale (CINSS)	1; (Orpana et al., 2019)
		The Satisfaction with Life Scale for Children	1; (Snowshoe et al., 2017)
		Child and Adolescent Mindfulness Measure (CAMM)	3; (Prenoveau et al., 2018; Guerra et al., 2019; ШУПЬГa, 2019)
Mindfulness/Spiritual	Ability to be present in life and the multidimensional concept that nurtures and celebrates wholeness through attention and awareness	Mindful Attention Awareness Scale	1; (Black et al., 2012)
		Spiritual wellbeing scale shortened version	2; (Cotton et al., 2005; Malinakova et al., 2017)
		Newly developed strong souls	1; (Thomas et al., 2010)
		Spiritual Wellbeing Questionnaire (SWBQ)	1; (Moodley et al., 2012)
		The FACIT-Sp-12 Spiritual Wellbeing Scale	1; (de Alvarenga et al., 2019)
Resilience	Ability to cope with and recover from adverse situations or stress	Child and Youth Resilience Measure (CYRM-28)	5; (Sanders et al., 2017; Panter-Brick et al., 2018; Slaten et al., 2019; van Rensburg et al., 2019; Kaunda-Khangamwa et al., 2020)
		Resilience Scale for Adolescents (READ)	4; (Hjemdal et al., 2006; Tratta et al., 2012; Kelly et al., 2017; Moksnes and Haugan, 2018)
		Chinese version of the resilience scale	1; (Shiah and Hwang, 2019)
		GMSR measure- gender minority stress and resilience scale	1; (Hidalgo et al., 2019)
		Modified Connor Davidson Resilience Scale	1; (Tomyn and Weinberg, 2018)
		Student Resilience Scale	1; (Casey et al., 2019)
		Student Resilience Survey	1; (Lereya et al., 2016)
Self-efficacy	Personal judgement on how well they will be able to cope in situations given the skills they possess	General Perceived Self-Efficacy Scale (GSE)	4; (Gabrielsen et al., 2012; Shoshani and Steinmetz, 2014; Royer-Gagnier et al., 2016; Ringdal et al., 2018)
		Adolescent self-consciousness questionnaire	1; (Espada et al., 2019)
		Emotional Self-Efficacy Instrumentation	1; (Valois and Zullig, 2013)
		Mandala Model of Self Scale (MMSS)	1; (Shiah and Hwang, 2019)
		Perceived Social Self-Efficacy (PSSE)	1; (Black et al., 2012)
		Research and action self-efficacy	1; (Ozer and Schotland, 2011)
		Self-Efficacy Scale	1; (Shiah and Hwang, 2019)
Self-esteem	Confidence in own abilities and worth	Rosenberg Self-esteem Scale (RSE)	15; (Ciarrochi et al., 2007; Hyun et al., 2009; Zhao et al., 2011; Armstrong, 2012; Meleddu et al., 2012; Toomey et al., 2013; Duy and Yildiz, 2014; Shoshani and Steinmetz, 2014; Eisman et al., 2016; Royer-Gagnier et al., 2016; St Clair et al., 2017; Canfield et al., 2018; Moksnes and Haugan, 2018; Ringdal et al., 2018; García-Álvarez and José Soler, 2019)
		Self-Compassion Scale (SCS)	2; (Gouveia et al., 2016; ШУПЬГa, 2019)
		The Self-Esteem Questionnaire (SEQ)	2; (Wild et al., 2005; Ozer and Schotland, 2011)
		Self-Perception Profile for children	1; (Donaldson and Ronan, 2006)
		Harter Self-Perception Profile for children	1; (Ey et al., 2005)
		Self-Compassion Scale- Short Form	1; (Abujaradeh et al., 2020)
		Tennessee self-concept scale	1; (Cavazos-Rehg et al., 2020)
Sense of Coherence	Ability to manage and cope with everyday life stressors due to their confidence and resources. A mixture of optimism and control	Sense of Coherence Scale (SOC-13)	1; (Gabrielsen et al., 2012)
		Orientation to Life Questionnaire	1; (Moksnes and Haugan, 2018)
		The Life Orientation Test-Revised (LOT-R)	1; (Shoshani and Steinmetz, 2014)
		The Social Capital and Cohesion Scale (SCCS)	1; (Magson et al., 2014)

Table 3 provides an overview of the summary characteristics of all the mental wellness instruments included in the qualitative synthesis.

**TABLE 3:** Mental wellness concepts by frequency of use and definitions.

Mental wellness concept	Frequency	Definition or Interpretation
Connectedness	7	Sense that one has satisfying relationships with others, believing that one is cared for, loved, esteemed, and valued, and providing friendship or support to others
Happiness	5	Steady states of positive mood and feeling content with one life, rather than momentary emotion.
Hope	4	Emotion characterised by positive feelings about the immediate or long-term future.
Life purpose/goal	4	You have goals in life and a sense of directedness; feel there is meaning to your present and past life; hold beliefs that give life purpose; and have aims and objectives for living.
Self-efficacy	4	A person’s particular set of beliefs that determine how well one can execute a plan of action in prospective situations. Self-efficacy is a person’s belief in their ability to succeed in a particular situation.
Personal expressiveness	4	Personal expressiveness and self-realisation are thus linked to eudaimonia, where what is considered worth desiring and having in life is the best within us or personal excellence. Experiences of an activity as personally expressive occur when there is (a) an unusually intense involvement in an undertaking, (b) a feeling of a special fit or meshing with an activity that is not characteristic of most daily tasks, (c) a feeling of intensely being alive, (d) a feeling of being complete or fulfilled while engaged in an activity, (e) an impression that this is what the person was meant to do, and (f) a feeling that this is who one really is
Life satisfaction	3	A person’s cognitive and affective evaluations of his or her life
Personal growth	3	You have a feeling of continued development; see yourself as growing and expanding; are open to new experiences; have the sense of realising your potential; see improvement in yourself and behaviour over time; are changing in ways that reflect more self-knowledge and effectiveness.
Autonomy	3	You are self-determining and independent; are able to resist social pressures to think and act in certain ways; regulate behaviour from within; and evaluate yourself by personal standards.
Physical functioning (feeling relaxed/energy)	3	Related to physical wellbeing, i.e., feeling relaxed, energy
Self-esteem	2	A person’s overall subjective sense of personal worth or value
Coping	2	Coping refers to cognitive and behavioural efforts to manage (master, reduce, or tolerate) a troubled person-environment relationship
Self-acceptance	2	A positive attitude toward yourself; acknowledge and accept multiple aspects of yourself including both good and bad qualities; and feel positive about your past life.
Environmental mastery	2	You have a sense of mastery and competence in managing the environment; control complex array of external activities; make effective use of surrounding opportunities; and are able to choose or create contexts suitable to your personal needs and values.
Engagement	2	Capacity to become absorbed in and focussed on what one is doing, as well as involvement and interest in life activities and tasks
Mindfulness/spiritual	1	Psychological process of bringing one’s attention to the internal and external experiences occurring in the present moment; concern for or sensitivity to things of the spirit or soul.
Sense of coherence	1	Degree of meaningfulness, comprehensibility, and manageability that people feel in their life
Self-control	1	The ability to control behaviours in order to avoid temptations and to achieve goals
Resilience	1	The ability to mentally withstand or adapt to uncertainty, challenges, and adversity.
Social contribution	1	Belief that one is a vital member of society, with something of value to give to the world. It includes the extent to which individuals believe that whatever they do in the world is valued by society and contributes to the common wealth
Social coherence	1	Concern for knowing about the world and constitutes the perception of the quality, organisation and operation of the social world. Social coherence involves appraisals that society is discernible, sensible and predictable.
Social actualisation	1	Similar to the themes of self-realisation and personal growth. Individuals with a high degree of social actualisation are hopeful about the condition and future of society, they recognise the potential that re- sides in a collective, and believe that the world can change and improve for people like themselves
Social acceptance	1	The social counterpart to self-acceptance and indicates that people hold favourable views of human nature, expect others to be capable of kindness and consequently feel comfortable with others
Perseverance	1	Ability to pursue one’s goals to completion, even in the face of obstacles.

### Mental Wellness Concepts From the General Mental Wellbeing Instruments

In line with the aims of this study, we examined each *Mental Wellbeing [general]* instrument to explore what mental wellness concepts are utilised to represent overall mental wellbeing. Through this process we were able to extract the individual mental wellness concepts within each of the seven Mental Wellbeing [general] instruments – which brought the total amount of mental wellness concepts to 24. In Table 3, we rank the 24 mental wellness concepts in order of frequency of use in the identified instruments.

### Discussion Definition of Mental Wellness Concepts

From our review, few authors (38%) explicitly defined the concepts of mental health, mental wellness or wellbeing as used in their study. This finding follows the trend in literature where mental wellness is not clearly or adequately defined and used interchangeably with mental health and mental wellbeing concepts (Ryan and Huta, 2009; Manderscheid et al., 2010; Ahanonu and Jooste, 2016; Fort Drum Regional Health Planning Organization, 2018; Witten et al., 2019; Gentzler et al., 2021). The observed absence of clear definitions or concepts of mental wellness, perpetuates the lethargy in adolescent research on mental wellness. This is an important point of consideration as ways in which concepts are defined affect the ways in which concepts are measured. Careful consideration should be taken on theory development and conceptualising mental wellness given the need for valid and reliable instruments specifically for adolescent populations. While instruments providing data on symptom and problem-oriented analyses are needed to improve our understanding of the mental health challenges adolescents face, there is a simultaneous need to examine positive aspects of mental health wellness to enhance our understanding of the different mental health related dimensions (Rose et al., 2017; Eriksson et al., 2019). This in turn, will be beneficial in aiding the development of theories and policies to guide sustainable health programmes which can address adolescent mental health on multiple levels of intervention and prevention.

The mental wellness concepts that were identified in this review are consistent with definitions of mental wellness in studies with adult populations (Ryan and Huta, 2009; Witten et al., 2019). However, the same fault line in interchanging mental wellness with mental wellbeing concepts is perpetuated. For example, Adams et al. (Adam et al., 1997) identified six dimensions of wellness – spiritual, physical, intellectual, emotional, psychological, and social – which provide a fitting categorisation for the wellness concepts identified from our review of mental wellness instruments for adolescents. Similarly, Ryff (Ryff et al., 2004) based her model of psychological wellbeing on concepts of self-acceptance, quality ties to others, a sense of autonomy, ability to manage complex environments to suit personal needs and values, pursuit of meaningful goals and purpose in life, and continued growth and development, – which are clearly synonymous with our mental wellness concepts for adults. This may suggest that frameworks for mental wellness developed for adult populations may be fitting for adolescents. However, this also indicates that the constraints encountered in the conceptualisation and operationalisation of mental wellness for research with adults are equally present in research with adolescents. Thus, the shortcomings in adolescent mental wellness research require critical conceptual development of mental wellness in general, with particular emphasis on reaching consensus on the definition of mental wellness, and the concomitant development of mental wellness indicators and measuring instruments. The mental wellness concepts identified from the instruments in this review may provide building blocks to conceptualise and guide theory development around adolescent mental wellness which can aid in the development of valid and reliable instruments.

Although definitions of mental wellness vary, the concepts of eudaimonia and hedonia are commonly agreed upon facets of mental wellness (Witten et al., 2019; Gentzler et al., 2021). From a *eudaemonic* perspective, mental wellness is associated with an individual’s ability to function well and reach their full potential/purpose in life, while the *hedonic* perspective associates mental wellness with positive affects (feeling well) in the present (Ryan and Deci, 2000; Eriksson et al., 2019; Gentzler et al., 2021). Research on eudaemonic and hedonic concepts have been well-documented for adult populations; indicating that both serve as protective factors against mental illness while promoting overall mental wellness (Witten et al., 2019; Gentzler et al., 2021). Witten et al. (2019) comment that research on hedonic mental wellness concepts in adolescents is well established, while less attention has been paid to eudaemonic concepts. To explore this, we categorised twelve of the thirteen types of mental wellness concepts to reflect eudaemonic (functioning well) and hedonic (feeling well) mental wellness. Our review identified six eudaemonic concepts, namely *coping, self-control, life purpose/goal, resilience, self-efficacy*, and *sense of coherence* from the existing mental wellness instruments. We also identified five hedonic concepts of *connectedness, happiness, hope, life satisfaction, and self-esteem* in this review. We argue that *mindfulness/spirituality* can be considered both a eudaimonic and hedonic concept depending on the context. Additionally, the seven *Mental Wellbeing [general] instruments* measured multiple mental wellness concepts, and therefore included both hedonic (*n* = 7) and eudaimonic concepts (*n* = 17). This suggests that instruments aimed at measuring general wellbeing as an indicator of adolescent mental wellness may reflect more comprehensive dimensions of mental wellness. In comparison to instruments which measure single concepts of mental wellness (i.e., self-esteem, resilience, etc.), we found that the general wellbeing instruments in this review provided a better representation of overall mental wellness.

In this review we see that hedonic mental wellness in adolescents goes beyond subjective feelings related to the self (i.e., happiness, hope, life satisfaction) but also includes social aspects centred on the adolescents’ connections to others and their sense of spirituality. While hedonic mental wellness is valuable, prioritising only happiness or life satisfaction may have unintended negative outcomes in adolescents. Therefore, a balance between eudaimonic and hedonic concepts is necessary to promote mental wellness. For example, research has shown that adolescents have less impulse control than adults and show heightened activation in brain regions associated with reward processing, meaning they tend to engage in reward-seeking behaviours and pursue hedonic pleasures more frequently, resulting in an imbalance in their mental wellness (Gentzler et al., 2021). Additionally, hedonic behaviours that are self-focussed (i.e., partying, self-indulgence) are related to negative affect, lower life satisfaction and more depressive symptoms (Gentzler et al., 2021). Therefore, exclusive emphasis on hedonic wellness may influence adolescents’ resilience and make them more vulnerable to social hardships and other challenges in life. In support of this, hedonic behaviours are considered less predictive of mental wellness than eudaemonic behaviours. As illustration, one longitudinal study of 15–17-year-olds found that hedonic decisions (gaining money for themselves) predicted greater depressive symptoms as opposed to eudaemonic behaviours (donating money) (Gentzler et al., 2021). Furthermore, emerging research suggesting that globally subjective wellbeing tends to decrease in young people between the ages of 13–24 years (Witten et al., 2019). The challenges related to the conceptualisation on mental wellness and related concepts and the lack of research focussing on both eudaimonic and hedonic mental wellness simultaneously makes it difficult to pinpoint the mechanism contributing to this phenomenon (Witten et al., 2019).

We argue that this decrease-with-age tendency may occur when adolescents only focus on pursuing hedonic mental wellness without developing eudaimonic mental wellness as the former is associated with lower levels of meaning in life (Witten et al., 2019). Therefore, while adolescents may experience happiness or life satisfaction, their overall subjective wellbeing may decline as they come to terms with getting older, increasing responsibilities, and making sense of the changing world around them. Without the balance of eudaimonic wellness, adolescents may struggle to cope with challenges and life demands associated with their development which could increase their engagement in risky behaviour as they try to stimulate the reward centre of their brains to maintain their hedonic wellness. On the other hand, adolescents who pursue both hedonic and eudaimonic mental wellness are more likely to adopt a balanced and realistic stance toward life, by accepting that challenges are a part of life while maintaining the belief that these are manageable, and that they are capable of working toward a better life. In such cases, the presence of eudaimonic wellness may act as a buffer against risks associated with hedonic pursuits. To support this, longitudinal studies have shown that a stabilising effect occurs as subjective wellbeing (SWB) scores stop decreasing over time (Casas and González-Carrasco, 2019). This stabilising effect may be influenced by the socio-political and cultural context in which adolescents develop – data shows that Australian adolescents SWB scores start increasing at ages 17/18, Brazilian adolescents SWB scores stop decreasing at 16, while data from Romania suggests SWB may continue decreasing after 20 years of age (Casas and González-Carrasco, 2019). Some cultures may provide more opportunities for adolescents to develop both hedonic and eudaimonic mental wellness, allowing them to regain a sense of SWB.

Additionally, due to the proliferation of quantitative studies aimed at measuring SWB in adolescents – we argue that more qualitative research is needed to explore why SWB decreases during these years, and which eudaemonic and hedonic factors may act as buffer to promote enduring mental wellness. Therefore, our review supports previous research indicating that both hedonic and eudaemonic indicators should be represented in instruments aimed at measuring mental wellness in adolescents – these indicators can be useful to track intervention and policy outcomes related to increasing adolescent mental wellness as it allows us to focus on gaining a better understanding of how they balance the desire to feel good and pursue a meaningful life, and under what conditions mental wellness is maintained.

Furthermore, while mental wellness instruments were originally developed for adult populations and adapted to demonstrate good reliability and validity scores among adolescents, it may be that these measures do not capture aspects of mental wellness that may be unique to adolescents (Laurenzi et al., 2020). As the decreasing-with-age tendency shows, some concepts related to mental wellness may be influenced by factors related to the developmental stage itself. This brings into question the extent to which research with adults can be generalised and adapted to adolescent populations. For example, the WEMWBS was originally developed to support work to develop Scottish mental health indicators for adults. This indicates that the identification and development of important mental wellness domains was derived from research focussing on aspects that are significant to adult mental wellness.

Similarities between adults and adolescent mental health exist. For example, research on mental health in adolescents living with HIV suggest that they experience similar challenges as adults living with HIV (Laurenzi et al., 2020). However, even in such cases there is a call to recognise the unique developmental stage adolescents are in as they experience significant mental and physical changes. Studies have shown that unlike adults, the adolescent brain is still developing and is more susceptible to changes caused by stress (Bodeker et al., 2020). Additionally, evidence shows that individual factors such as age, sex, and gender present complex interactions with mental wellness among adolescents (Bodeker et al., 2020). Therefore, there is a need to conduct more research with adolescents so that they may participate and contribute to the conceptualisation and operationalisation of mental wellness which suits their needs.

Additionally, we found that all the instruments were originally developed in English, with many of these being developed with participants from western context (see Figure 2). The exception being the Urban Adolescent Hope Scale and the Strong Souls instruments which were developed with indigenous Australian Youth. While many of these instruments have been translated to other languages and validated in different cultural contexts, it raises questions which align with debates around decolonising mental health such as what constitutes as knowledge and “evidence” of global mental health and who decides what counts as “evidence” (Fernando and Moodley, 2018; Horn, 2020; Morton et al., 2020).

Mental wellness measures which are developed in western contexts are often translated and validated in other cultural settings as developing new measures are seen as involving costly and time-consuming processes (Mills, 2014). However, do these validated measures accurately capture the evidence related to mental wellness experiences and challenges of youth living in cultural and religious contexts that differ from the west, or are they perpetuating definitions based on Western Psychology? According to Zaretsky (2021), mental healthcare systems continue to be shaped by colonial policies and practices rooted in racism which consequently results in the perpetuation of mental wellness services that are not culturally appropriate.

Research shows that there are mental health disparities between indigenous and non-indigenous youth resulting from centuries of racism and colonial practices (Gould et al., 2020; Morton et al., 2020; Zaretsky, 2021). Indigenous youth are more likely to experience trauma, suicide attempts, substance use, HIV, homelessness, and mental health problems than non-indigenous youth (Gould et al., 2020; Morton et al., 2020). Furthermore, treating mental health from a western perspective can perpetuate colonial and oppressive practices (Gould et al., 2020). Indigenous youth are being hospitalised at a higher rate than non-indigenous youth, and the treatment involved may increase their chances of developing substance dependence (Morton et al., 2020). Studies like these suggest that indigenous adolescents and those living outside of western context may experience challenges to their mental health which differ from non-indigenous youth. This also means that the strategies they need to improve and maintain their mental wellness could take on forms which are generally not considered in the western context. Therefore, understanding what mental health wellness is from a decolonised perspective is necessary to increase the recognition of indigenous approaches to healing which should be incorporated into mental health services.

### Study Limitations and Future Research

There are few limits to note regarding this study. Firstly, the databases we used to search may not have accessed psychology relevant databases. As mentioned previously, some of the articles were excluded from this review as we did not have access to those via the university databases. Based on the results of our review, and the global advocacy around adolescents’ rights, we argue that there is a need for more qualitative research to explore how adolescents experience and understand mental wellness. This research can be used to address questions around how adolescents make sense of mental wellness, what are their experiences and interpretations of wellness and what do they need to improve and maintain mental wellness. These are critical questions which require further investigation as adolescents understanding of the concept during this unique developmental period, may influence their lifestyle choices and behaviours, which consequently may be carried into adulthood. This information is vital to support the development of mental health services which are accessible and valuable to adolescents. Most instruments in this review were designed in developed countries. Cross-cultural qualitative studies may help us explore how adolescents from different cultures experience mental wellness, which may help us to better understand the need to develop instruments in developing countries. Additionally, qualitative research exploring adolescents view of mental wellness may provide further insights to the similarities or differences between adolescent and adult populations, which can facilitate the development of instruments that specifically measure adolescent mental wellness.

## Conclusion

The review confirms that there is a growing body of literature on adolescent’s mental health. However, there is no consensus on an explicit definition for the concept of mental wellness. A clear definition is needed to improve our understanding about adolescent mental wellness specifically, which can aid the development and monitoring, and evaluation of interventions and programmes aimed at improving adolescent mental wellness. Our findings indicate that the general mental wellbeing instruments reflected a more comprehensive measure of mental wellness, highlighting the need for the inclusion of both hedonic and eudaemonic indicators in mental wellness measures for adolescents. A key finding is the adaptation of the instruments to adolescent populations as most instruments were developed for adults in English language and in a developed context. This indicates a need for adolescents to be involved in the conceptualisation and operationalisation of mental wellness. Mental wellness instruments should allow for the varied presentation of mental wellness evident amongst youth in different cultural contexts.

## Author Contributions

ZO contributed to the conceptualisation and management of the review process, fieldwork, data extraction, provided leadership and input to the review team at each stage of the project, and the conceptualisation of the manuscript. FM contributed to devising an operational plan for fieldwork, refinement of the review process, coordination of the fieldwork and data extraction. ZO and FM contributed to the drafting, technical aspects and critical revisions of the article and approved the submitted version. BV contributed to the conceptualisation of the review, fieldwork, draft write up, revisions and editing of the manuscript, provided leadership and input to the review team at each stage of the project, contributed to the conceptualisation, technical aspects, and critical revisions of the manuscript and approved the submitted version. All authors contributed to the article and approved the submitted version.

## Conflict of Interest

The authors declare that the research was conducted in the absence of any commercial or financial relationships that could be construed as a potential conflict of interest.

## Publisher’s Note

All claims expressed in this article are solely those of the authors and do not necessarily represent those of their affiliated organizations, or those of the publisher, the editors and the reviewers. Any product that may be evaluated in this article, or claim that may be made by its manufacturer, is not guaranteed or endorsed by the publisher.
